# Sulfonal

**Published:** 1888-11

**Authors:** 


Sulfonal.—This new hypnotic is being tested clinically in a variety of ways, by competent physiologists and clinicians. We hope to present our readers with some observations next month, made by reliable authorities. Messrs. W. H. Schieffelin & Co., New York, furnish the drug in pure and convenient form for administration.

